# Right ventricular septal pacing- clinical and electrical predictors for LV contraction asynchrony

**Published:** 2014

**Authors:** C Iorgulescu, DA Radu, D Constantinescu, C Caldararu, M Dorobantu

**Affiliations:** * Bucharest Clinical Emergency Hospital, University of Medicine Carol Davila; ** Bucharest Clinical Emergency Hospital; *** Monza Hospital, Bucharest

## Abstract

Purpose: Prolonged pacing from the right ventricular apex (RV) is associated with the LV dyssynchrony leading to progressive left ventricular dysfunction and increased morbidity and mortality. Alternate RV pacing sites-in particular the mid- RV septum and the RV outflow tract (RVOT) septum were considered, but no clear benefit was proven till now for this pacing sites. This may be due to the heterogeneity of the RV septal positions and to the significant number of leads placed on the RV free wall.

The aim of this study is to find a reliable method of septal lead placement and to identify those pacing sites which provide better LV electrical activation

Methods: 50 consecutive patients referred for pacemaker implants due to AV block were included. Patients with history of heart failure or LVEF < 50% at the implant were excluded. All patients had RV leads placed in septal position. This was achieved with a double curved stilet with the distal curve aimed posteriorly. RV septum and RVOT were mapped during implant aiming for a narrow paced QRS with an axis as close to normal as possible. Pacing lead position was evaluated during the implant using fluoroscopy (AP and LAO 40 °) and than by 12 lead ECG and echo. IntraLV dyssynchrony was evaluated during pacing using SPWMD in short axis parasternal view and the TDI septal to lateral ∆ t. Paced QRS duration and axis were also recorded. The correlation was sought between lead position evaluated by Rx and by echo and between paced QRS duration and axis and LV dyssynchrony.

Results: 92%(46) of the patients had the lead in septal position RV (32 in the mid-septal RV and 14 in RVOT), while 8% (4 pts) had the lead on the RVOT RV free wall as shown by echo. An anterior-oriented lead in the left anterior oblique fluoroscopic projection was specific for free wall position while positive QRS in DI in RVOT position was suggestive for free wall position on the ECG.

No correlation was made between paced QRS axis and LV dyssynchrony while the QRS duration of > 160 ms was associated with significant LV dyssynchrony (SPWMD > 130 ms and to lateral septal ∆ t > 70 ms).

Conclusions: RV lead placement on the RV septum can be reliably achieved using a specially curved stilet and the LAO projection for confirmation.

The wide paced QRS is correlated with significant intra LV dyssynchrony and therefore the RV pacing site with the narrowest QRS should be sought.

## Premises:

Stimulation of the right ventricle apex (RVA) leads to an abnormal electrical activation of ventricular myocardium - starting from the apex towards the base and from the right to the left. This increases the total ventricular activation time and delays the contraction of the lateral wall of LV. Over the long term the RVA pacing is associated with systolic dysfunction of LV, heart failure, increased incidence of atrial fibrillation and increased mortality. Pathophysiological mechanisms proposed to explain these phenomena are the dyssynchrony of contraction of LV, tampering with the cardiac hemodynamics, increased myocardial oxygen consumption and workload of the LV, followed by both structural alterations at the cellular level and macroscopical remodeling of LV [**1**-**4**]. Alternative positions have been proposed for the pacing leads, including the right ventricular outflow tract (RVOT), basal and mid-interventricular septum as the most often used [**5**-**8**]. These positions aim to obtaining a narrower paced QRS with an electrical axis closer to normal compared to the one obtained by stimulating RVA, but have not shown a consistent benefit for LV activation dyssynchrony or LV systolic dysfunction incidence in clinical trials [**9**,**10**] . This may be due to heterogeneity of septal ventricular pacing lead positions and implantation of a significant percentage of leads in the free wall of the RV by using the classical methods of implant [**11**,**12**].

The aim of this study is to achieve effective methods for intraprocedural identifying of septal positions which ensure optimum electrical activation of the left ventricle.

## Methods

**Study group**

We included in the study 50 consecutive patients with atrioventricular block referred for permanent cardiac pacemaker implantation. Patients with LV ejection fraction < 40% and those with a history of heart failure were excluded. The medium age was 74 ± 12 years.

Of the 50 patients 27 were males (54%), 39 were in sinus rhythm and 11 in atrial fibrillation.

Associated comorbidities were as follows: 34 patients (68%) were hypertensive, 11(22%) had ischemic heart disease (documented prior coronary events), 9(18%) were diabetic and 31(62%) were dyslipidemic. All patients had normal or slightly impaired systolic function -the average left ventricular ejection fraction was 52%, ranging from 45% to 60%.

Pacing indication was complete AV block in 26 patients, 2nd degree AV block in 16 patients and bundle branch block with intermittent AV block in 8 patients.

Patient characteristics are listed below in (**Table 1**)

**Table 1:** Patients characteristics

Age	74.14 ±16
Sex	27 (54%) males
HBP	34 (68%)
IHD	11 (22%)
DM	9 (18)
Hyperlipemia	31 (62%)
LVEF	52±7%
Baseline QRS duration	135.6 ±38 ms

**Implant technique**

**Fig. 1:**
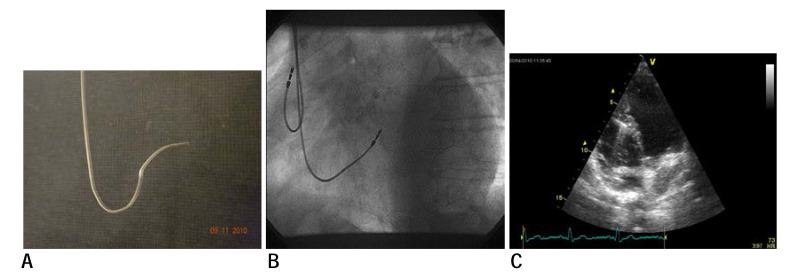
A- double curved stilet used for RV septal lead placement; B- LAO fluoroscopic incidence of a septal RV lead; C- apical 4 chamber view showing septal placement of RV lead.

Pacemaker implant was performed under local anesthesia with Lidocaine 1%. The favorite approach was the cephalic vein (44 patients) or subclavian vein (6 patients). Active fixation leads were used for implant -Capsurefix Novus -Medtronic, Tendril-StJude-and Setrox - Biotronik. In all patients placement of the RV lead in a septal position was attempted. To obtain this position we preferred the technique described by Mond and [**13**] coworkers-using a dual-curve stilet: a large proximal upward curve and a distal acute curve aimed posterior- (see **Fig. 1 A**). The stilets were the standard straight ones provided by the manufacturer along with the pacing leads, and they were modeled intraprocedural. The size and angulation of the stilet curves was left to the discretion of the operator.

The implant was performed under fluoroscopic guidance using the postero-anterior (PA) and left anterior oblique (LAO) incidence. Right ventricle lead was positioned at the septum or outflow tract by checking the duration, axis, pacing threshold and sensing in every position and trying to get a narrow paced QRS, with an axis as close as possible to normal, while not compromising the pacing and sensing of the lead. Septal positioning has been checked at the end of the implant using LAO fluoroscopic incidence(**Fig. 1 B**). The leads targeted posterior in this incidence were considered to be positioned on the interventricular septum, while the anterior-oriented leads were considered to be on the RV free wall and were repositioned.

**Post implant evaluation**

All patients performed post implant standard 12 leads ECG, with 25 mm/s paper speed and calibration of 10 mm/mV. QRS duration and QRS axis were measured during RV pacing.

In all patients was performed transthoracic echocardiography - using a General Electronics VIVID 7. The position of the lead on the interventricular septum or on the free wall of the right ventricle was appreciated in apical four chamber (**figure 1 C**) and parasternal short axis views. 

We evaluated the presence of intraventricular LV dyssynchrony during ventricular pacing by measuring the time interval from the peak of contraction of the interventricular septum (IVS) to the peak of contraction of the LV posterior wall (septal to posterior wall motion to delay-SPWMD) in M mode parasternal short axis incidence at the level of the papillary muscles and by tissue doppler imaging – the time delay between the peak of the contraction curves of the IVS and the lateral wall- septal to lateral ∆ t. Intra LV dyssynchrony was considered to be present if the patient had SPWMD > 130 ms and/or septal to lateral ∆ t-> 70 ms.

**Statistical methods**

The variables in the database were described by number of cases (n), and percentages (%), in case of discrete variables and means (m) and interquartile range in case of continuous variables. 

The statistical analysis was performed after dividing the patients between “Without dyssynchrony” and “With dyssynchrony” according to the value of a supplementary variable “Diss”. Diss was considered to be positive if either SPWMD (septal to posterior wall motion delay) measured in short axis or TDISL (septal to lateral delay through tissue Doppler) were positive. When discrete variables were analyzed by these subgroups, either Fisher’s or χ2 test were used, according to the number of subgroups in the cross tabulation (Fisher for only two subgroups of each variable, χ2 in case that any of the variables had more than two groups). In case a continuous variable was analyzed according to the subgroups, ANOVA test was used in order to point out statistically significant difference between the means. A p value of 0.05 was considered relevant in both cases; were necessary 95% CI were also mentioned. 

Supplementary, a cutoff for significant QRS width in patients with dyssynchrony positive by either SPWMD_ShortAxis or TDISL was searched. This was achieved by coding eight supplementary variables V1 through V8, considered to be positive if QRS width was >130 msec, >140 msec,… >200 msec consecutively. Afterwards, a Fisher’s test between two discrete variables were performed. Surprisingly, a statistically significant p-value was obtained starting from a cutoff of 140 msec of QRS Width. However, a scatterplot analysis revealed that the least superposition of wide QRS patients with dyssynchrony and without dyssynchrony occurred after 160 msec, therefore this value was chosen as a sensitive and specific cutoff.

## Results

Positioning the RV lead was carried out successfully in all 50 cases without intraprocedural complications. They had been five intraprocedural repositions, based on the anterior orientation in the left oblique incidence. Electric parameters were excellent with a capture threshold of 0.65 V/0.4 ms (with values from 0, 25V to 1.25 V) and an average of 8.7 mV detection (with values between 4.5 and 15 mV). Fluoroscopy time for positioning the RV lead was averaging 1.5 min with variations between < 1 min and 4 min. Post procedural complications consisted of 2 local hematoma treated conservatively. There were no lead depositions, infections or perforations for a follow up period of 6 months on average (between 1 and 12 months).

According to echocardiographic patterns 46 (92%) patients had the RV lead in a septal position- 32 at midseptal level and 14 in the RVOT -and 4 patients had the RV lead on the RV free wall . Significant intraLV dyssynchrony (> 130 ms SPWMD and TDI septal to lateral ∆ t-> 70 ms) was found in 19 patients (36%) (**Table 2**).

All patients had a paced QRS with LBB morphology and inferior electrical axis, characteristic of right ventricular septal pacing. Average duration of paced QRS was 155 ± 35 ms. The duration of the paced QRS was greater in the case of RV free wall positions -175 ± 10ms, compared with true septal positions – 153 ± 33ms (**Table 3**).

Multivariate analysis between group A (without dyssynchrony) and group B (with dyssynchrony) showed a statistically significant correlation for age, paced QRS duration and RV free wall position (**Table 2**). No significant difference was found for sex, hypertension, ischemic heart disease, diabetes and hyperlipemia, as well as for baseline QRS duration and for paced QRS axis. LV ejection fraction was lower in the dyssynchrony group, reaching statistical significance.

**Fig. 2:**
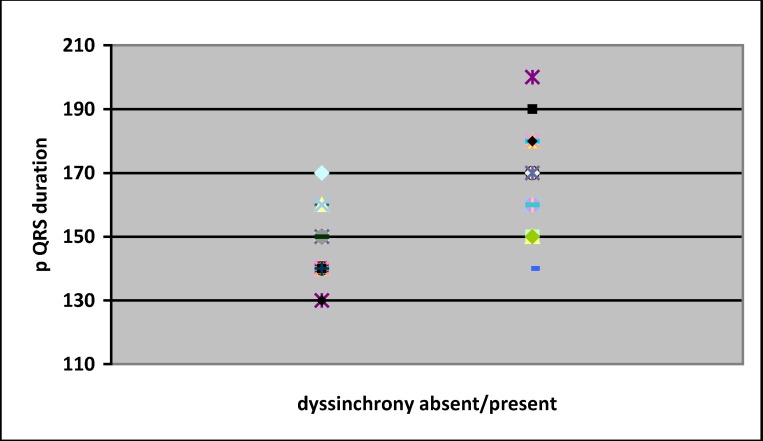
Graphic representation of QRS duration in the two groups

A “cloud” representation of QRS duration of the two groups was made – (**Fig. 2**). Based on this a cut-off value of 160 ms was chosen, as to provide a good specificity-sensitivity ratio.

**Fig. 3:**
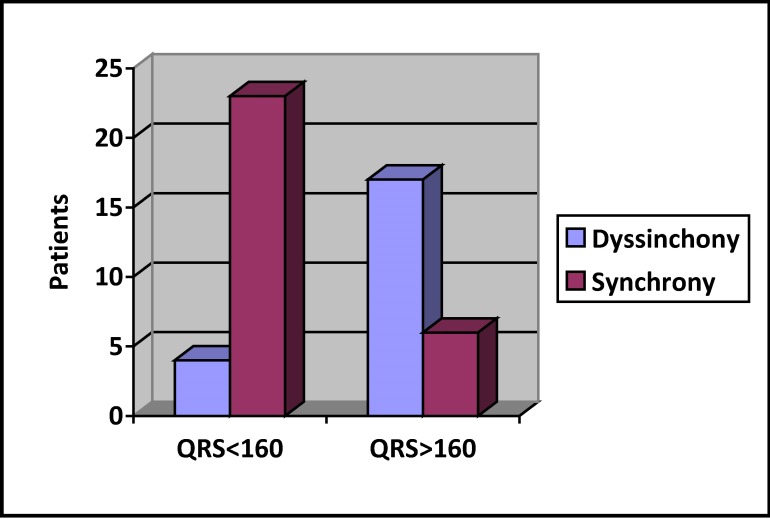
Presence of LV dyssynchrony based on QRS duration

Paced QRS duration ≥ 160 ms was statistically significant associated with the presence of intra LV dyssynchrony, with a 74% sensitivity and 85% specificity, P < 0,000 – (**Fig. 3**). Paced QRS axis did not influence the presence of intraLV dyssynchrony.

**Table 2:** Statistical significance of different variables between the two study groups.

	Group A without dyssinchrony *	Group B with dyssinchrony**	p a	95% CI
No#	31	19	-	-
Sex (Females), n (%)	11 (35.48%)	12 (63.15%)	0.081	-
Age (Years), m (IQR)	72.55 (6, 69-75)	76.64 (7, 74.5-81.5)	0.035	0.035 * - (70.39-74.70); ** - (73.01-80.46)
Implant indication (CAVB n(%); 2ndAVB n(%); BBB n(%))	13 (41.93%); 13 (41.93%); 5 (16.12%)	11 (57.89%); 5 (26.31%); 3 (15.78%)	0.491	-
Hypertensive (Yes), n (%)	23 (74.19%)	11 (57.89%)	0.349	-
Ischemic (Yes), n (%)	4 (12.9%)	7 (36.84%)	0.078	-
DM (Yes), n (%)	3 (9.67%)	6 (31.57%)	0.063	-
Dyslipidemic (Yes), n (%)	18 (58.06%)	13 (68.42%)	0.556	-
LVEF (%), m (IQR)	53.55 (5, 50-55)	50.00 (0, 50-50)	0.000	* - (52.47-54.63); ** - (48.20-51.80)
NativeQRSWidth (Msec), m (IQR)	130.65 (30, 110-140)	143.68 (20, 140-160)	0.061	* - (122.78-138.51); ** - (131.11-156.25)
StimQRSWidth (Msec), m (IQR)	148.06 (10, 140-150)	166.84 (30, 150-180)	0.000	* - (144.60-151.53); ** - (159.30-174.39)
StimQRSAxis (Degrees), m (IQR)	51.29 (15, 45-60)	44.21 (30, 30-60)	0.377	* - (41.37-61.21); ** - (30.91-57.51)
RVLeadPosition (septal n(%)vs free wall n(%))	31 (100%); 0 (0%)	15 (78.94%); 4 (21.05%)	0.017	-
*- with both SPWMD (short axis view measurement) and TDISL negative; **- with either SPWMD or TDISL positive				
IQR = interquartile range				
Two-sided p values were calculated using the χ2 test (for ≥ 3 groups) and Fisher’s exact test (for two groups) for categorical variables and using the ANOVA test for continuous variables				

**Table 3:** Results: paced QRS duration, QRS axis and the presence of intraLV dyssynchrony depending on the RV lead position on the IVS or the free wall of RV.

	IVS	Free wall
RV lead position	46 patients	4 patients
Paced QRS duration	153ms	175ms
Paced QRS axis	35 °	55 °
SPWMD > 130 ms and/or ∆ t septo-lateral-> 70 ms	15 patients	3 patients

## Discussion

Septal positioning of the RV lead was obtained with a high success rate using a double curve stilet and PA radiological incidence - reaching a real septal position in 82% of the patients. The incidence of LAO provides additional information in confirmation of a septal position, with a remaining 8% percent of the leads positioned on the RV free wall even after using this incidence. This success rate of septal position confirms that of 90% described by Mond [**13**] and is superior to those obtained using single curve stilet - 61% [**12**]. The rate of complications was minimal, the electrical parameters were excellent, the fluoroscopy time was acceptable.

The electrocardiographic characteristics of paced QRS obtained during the implant seem useful for choosing the optimal position of the RV lead -presence of a paced QRS complex broader than 160 ms indicating a position in which RV pacing will most likely produce significant LV dyssynchrony. This is due to the correlation between the duration of the QRS and delayed ventricular activation of the lateral wall of the LV in the event of increased ventricular activation time. Therefore in the case of obtaining a broader QRS complex than 160 ms in one particular position of the lead of RV is preferable to change this position as to get a narrower paced QRS. It is known from the MOST study14 that QRS duration correlates with an increased incidence of heart failure. Dyssinchronous LV activation leads to LV remodeling , the appearance of heart failure and increased mortality. Getting a wide paced QRS indicates increased risk for all of them. 

IntraLV dyssynchrony remains a significant presence in our study even in the case of septal stimulation-35% of patients. Using the paced QRS duration for the choice of pacing site might help reducing this percentage. Paced QRS axis did not proved useful in the case of septal pacing RV for prediction of intraLV asynchrony. This is probably due to the fact that the duration of the paced QRS depends on capturing the His-Purkinje system and the place where you can get this may be situated in a higher or lower septal position depending on the AV block site – paced QRS axis is situated more vertically or horizontally depending on the exact location of the lead of RVwith an axis close to 90 ° in case of ejection tract of RV and closer to 0 ° in the case of mid-septal positions.

QRS complex duration was larger in the case of RV free wall positioning. All these patients have had higher LV asynchrony and QRS durations. In fact free wall placement of the RV lead had the strongest statistical association with intraLV dyssynchrony in our study. This arguments against positioning the RV lead on the free wall, but the study lacks the number of RV free wall pacing cases needed in order to reach this conclusion .

**Fig. 4:**
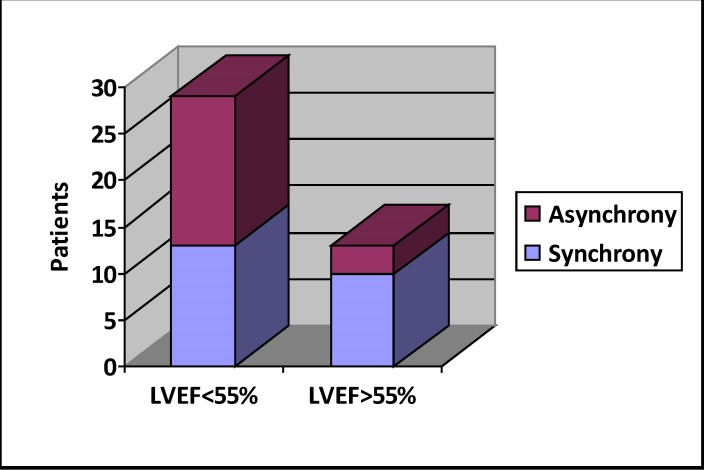
Asynchrony incidence in normal and impaired systolic function subgroups

Patients in group B had lower ejection fraction – 50% vs 53,5%, p<0,05. Patients with slightly impaired systolic function had higher incidence of LV asynchrony – (**Fig. 4**). This finding leads to the idea of an increased risk of pacing induced cardiomyopathy, warranting further research in this direction and also a careful follow up for this subgroup of patients.

**Fig. 5:**
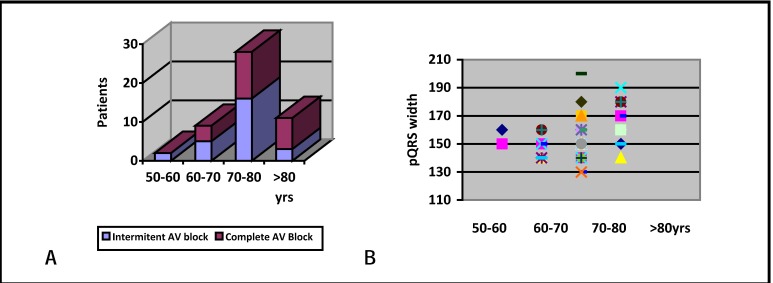
A -Age related incidence of complete AV block, B – Age related distribution of QRS width

Age correlated significantly with LV asynchrony in our study. Although this could be a coincidental finding in a small study group, it could also mean that age is associated with more severe conduction sistem disease, as would be sugested by higher incidence of complete AV block and wider paced QRS complexes in these patients – (**Fig. 5**). Severe His-Purkinje disease would mean a more delayed LV lateral wall contraction, resulting in intra LV assynchrony.

Even if sex did not reach statistical significance, we notice the presence of 63% females in group B, compared with 36% in group A. A larger study sample could provide a statistical power to reach the conclusion the female sex is associated with LV assynchrony during RV pacing. This would correlate well with the fact that females with left bundle branch block and dilative cardiomyopathy are better responders to cardiac resinchronisation theraphy [**15**].

## Conclusions

1. Positioning the pacing lead on the RV septum can be performed accurately using a double curve stilet with a proximal upward curve and a distal posterior curve, using 40° LAO radiological incidence for confirmation.

2. Paced QRS duration > 160 ms is significantly associated with presence of intraLV assynchrony, indicating that during the implant must be sought the position with narrowest paced QRS. 

3. The axis of the QRS does not influence the assynchronous contraction of LV and should not be used to optimize the position of the RV lead.

4. Positions at the level of the RV wall free are associated with a broader QRS and LV assynchrony and should be avoided.

5. Age correlates with LV asynchrony during ventricular pacing, so seeking a narrower paced QRS might be even more important in older people.

6. Patients with slightly impaired systolic function tend to have more frequently LV dyssynchrony. Echocardiographical follow-up would be recommended in this subset of patients, as to early detect pacing induced cardiomyopathy and promptly provide upgrade to a biventricular system.

**Limitations**

The major flaws of this study are its transversal design and the relatively small sample size. However the purpose of this research was not to definitely ascertain the risk factors for pacing induced cardiomyopathy but to detect the potential directions which warrant further investigation in this quite frequent and relatively unexplored pathology.
